# Taking Cold

**Published:** 1872-04

**Authors:** 


### TAKING COLD.

A LADY of wealth was at an evening party; the carriage was not at the door when she was ready to return, and rather than wait, she proposed to her husband to walk home, as it was but a short distance; there was a slight fall of snow on the pavement, and she had on thin slippers. The next morning she had a cold, seldom left her bed for four years, and shortly after died of consumption. Her father, mother, and grandparents had died at the ages of four and five score.

Longevity is to a certain extent inherited, but exposure or abuse of the constitution, bad habits of life, excessive indulgence in the appetites and passions will overbalance the advantages of a vigorous constitution.

There are many who feel so well and strong that they appear to think that nothing can hurt them. The history of such is often ended in the announcement in the morning papers, "Died suddenly, yesterday." While multitudes of those who seem so frail that a little wind would blow them over, reach a good old age, because they feel compelled to take care of themselves all the time, and instinctively avoid exposures.

